# Supplementary material for: Hydroxy-3-Phenylcoumarins as Multitarget Compounds for Skin Aging Diseases: Synthesis, Molecular Docking and Tyrosinase, Elastase, Collagenase and Hyaluronidase Inhibition, and Sun Protection Factor
Source: Molecules. 2022 Oct 15;27(20):6914. doi: 10.3390/molecules27206914 (PMC9611449; doi:10.3390/molecules27206914)
Supplement: Supplementary file 1 [file molecules-27-06914-s001.zip › molecules-1942532-supplementary.pdf]

Supplementary Materials

Hydroxy-3-Phenylcoumarins as Multitarget Compounds for Skin Aging Diseases: Synthesis, Molecular Docking and Tyrosinase, Elastase, Collagenase and Hyaluronidase Inhibition, and Sun Protection Factor

Francesca Pintus [1,†], Sonia Floris [1,†], Antonella Fais [1,*], Benedetta Era [1], Amit Kumar [2], Gianluca Gatto [2], Eugenio Uriarte [3,4] and Maria João Matos [3,*]

[1] Department of Life and Environmental Sciences, University of Cagliari, 09042 Monserrato, Italy; fpintus@unica.it (F.P.); s.floris@unica.it (S.F.); era@unica.it (B.E.)
[2] Department of Electrical and Electronic Engineering, University of Cagliari, 09123 Cagliari, Italy; amit369@gmail.com (A.K.); gatto@unica.it (G.G.)
[3] Departamento de Química Orgánica, Facultade de Farmacia, Universidade Santiago de Compostela, 15782 Santiago de Compostela, Spain; eugenio.uriarte@usc.es
[4] Instituto de Ciencias Químicas Aplicadas, Universidad Autónoma de Chile, 7500912 Santiago, Chile
* Correspondence: fais@unica.it (A.F.); mariajoao.correiapinto@usc.es (M.J.M.)
† These authors contributed equally to this work.

Content:

1. **Figure S1.** ^{1}H NMR and ^{13}C NMR spectra of the new compounds **2-8**, **10** and **12**.
2. **Table S1.** Inhibition percentage of compounds against tyrosinase, elastase, collagenase and hyaluronidase.

Compound 2

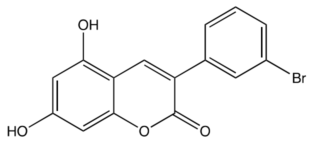
OH
Br
HO
O
O

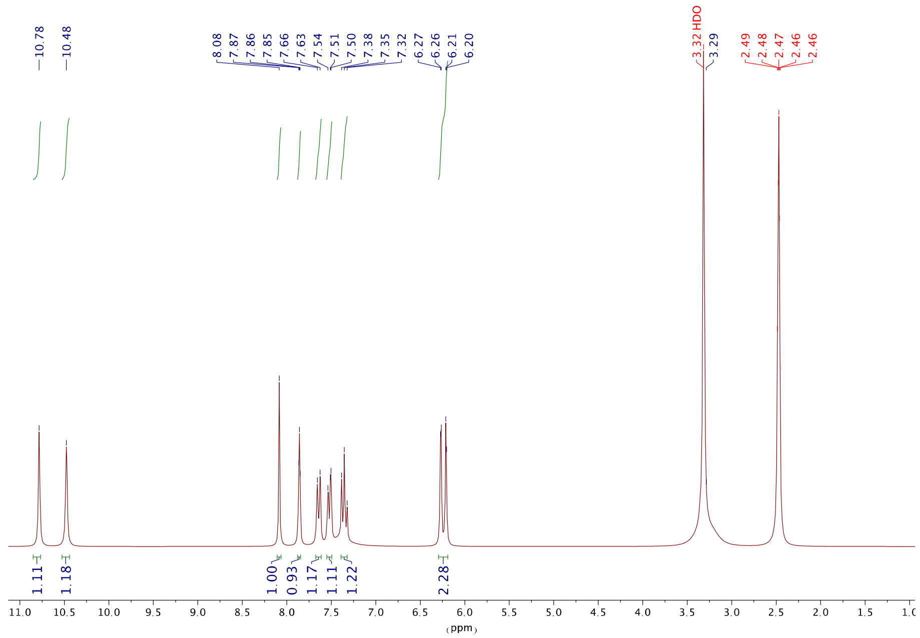
10.78
10.48
8.08
7.87
7.86
7.85
7.66
7.63
7.54
7.51
7.50
7.38
7.35
7.32
6.27
6.26
6.21
6.20
3.32 HDO
3.29
2.49
2.48
2.47
2.46
2.46
1.11
1.18
1.00
0.93
1.17
1.11
1.22
2.28
(ppm)

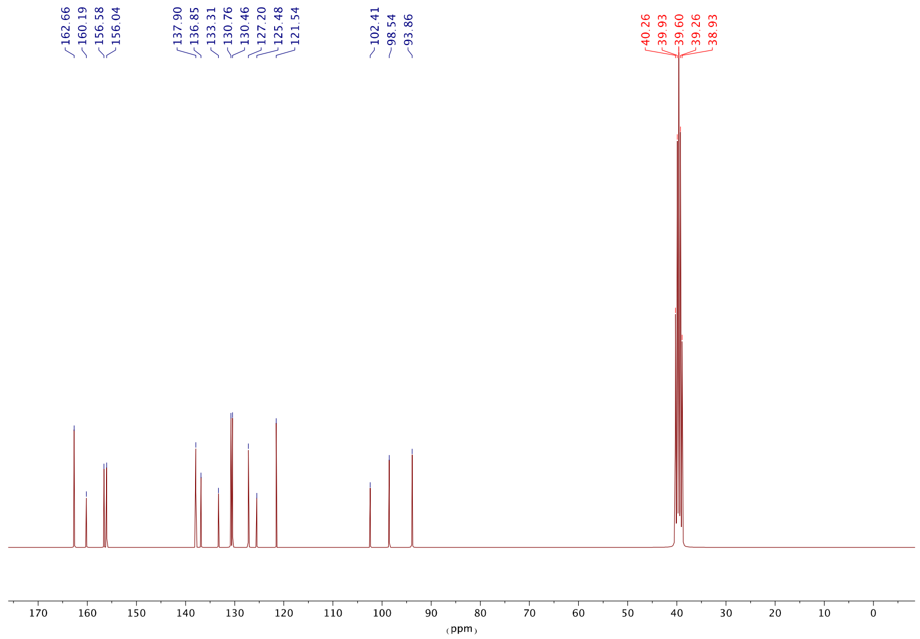
162.66
160.19
156.58
156.04
137.90
136.85
133.31
130.76
130.46
127.20
125.48
121.54
102.41
98.54
93.86
40.26
39.93
39.60
39.26
38.93
(ppm)

Compound 3

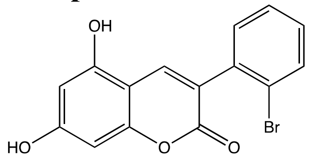
OH
HO
O
O
Br

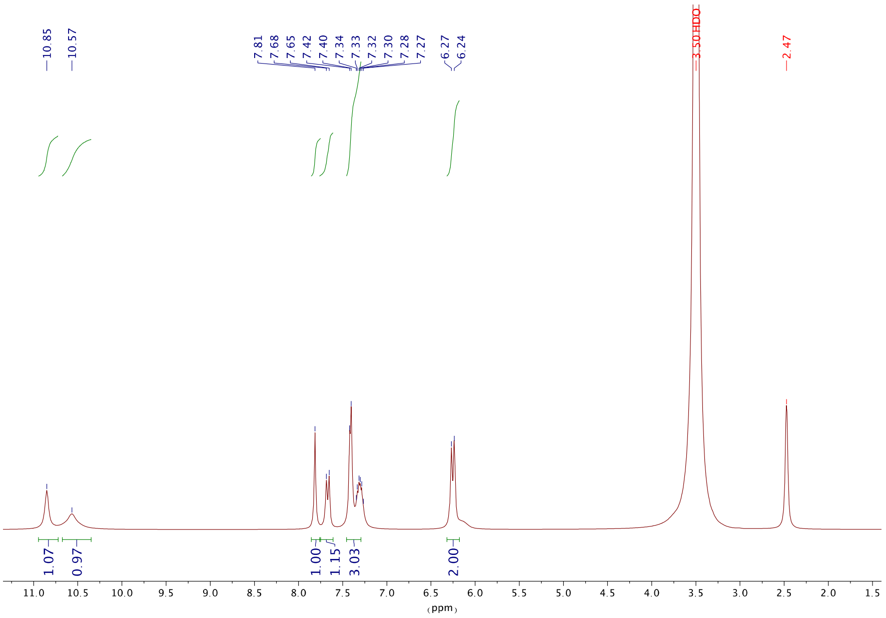
10.85
10.57
7.81
7.68
7.65
7.42
7.40
7.34
7.33
7.32
7.30
7.28
7.27
6.27
6.24
3.50 HDO
2.47
1.07
0.97
1.00
1.15
3.03
2.00
(ppm)

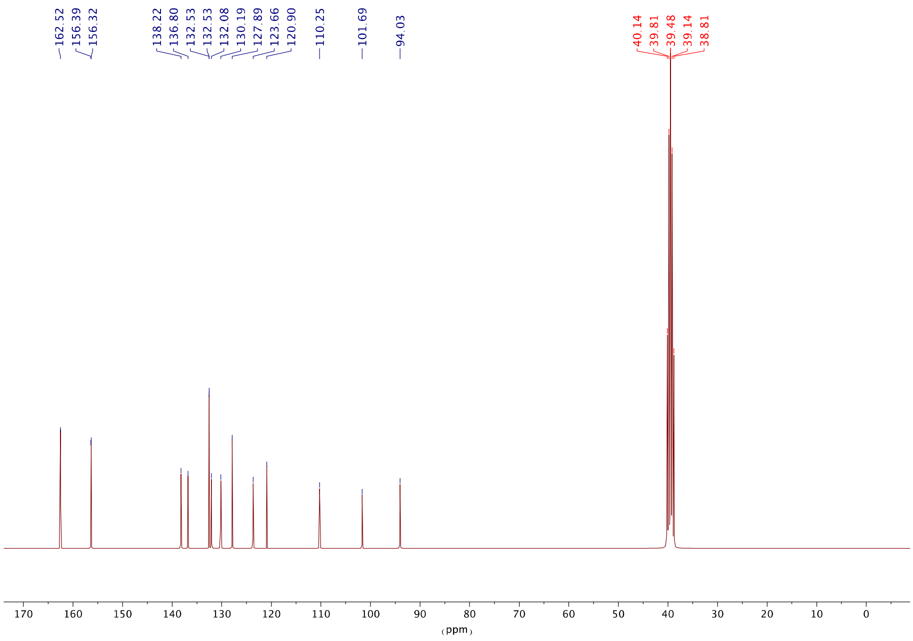
162.52
156.39
156.32
138.22
136.80
132.53
132.53
132.08
130.19
127.89
123.66
120.90
110.25
101.69
94.03
40.14
39.81
39.48
39.14
38.81
(ppm)

Compound 4

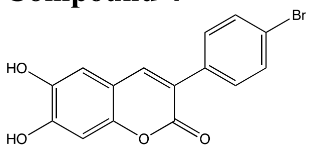
Br
HO
HO
O
O

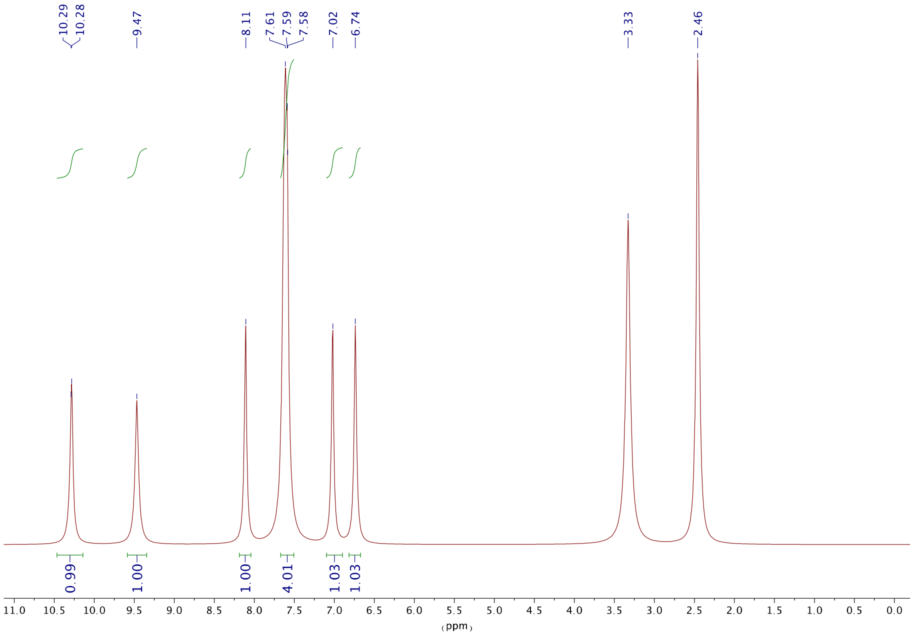
10.29
10.28
9.47
8.11
7.61
7.59
7.58
7.02
6.74
3.33
2.46
0.99
1.00
1.00
4.01
1.03
1.03
11.0 10.5 10.0 9.5 9.0 8.5 8.0 7.5 7.0 6.5 6.0 5.5 5.0 4.5 4.0 3.5 3.0 2.5 2.0 1.5 1.0 0.5 0.0
(ppm)

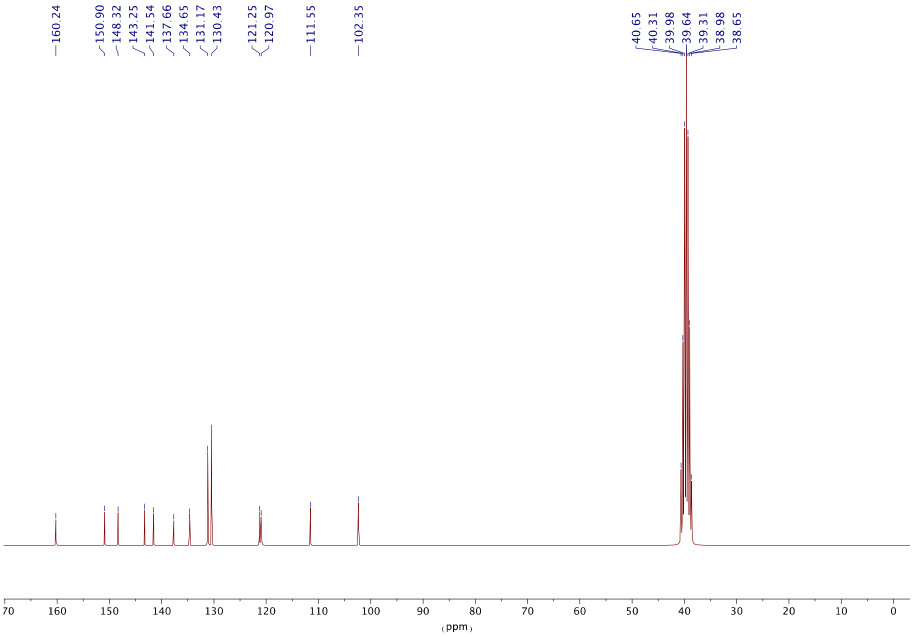
160.24
150.90
148.32
143.25
141.54
137.66
134.65
131.17
130.43
121.25
120.97
111.55
102.35
40.65
40.31
39.98
39.64
39.31
38.98
38.65
70 160 150 140 130 120 110 100 90 80 70 60 50 40 30 20 10 0
(ppm)

Compound 5

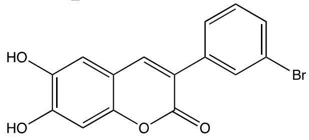
HO
HO
O
O
Br

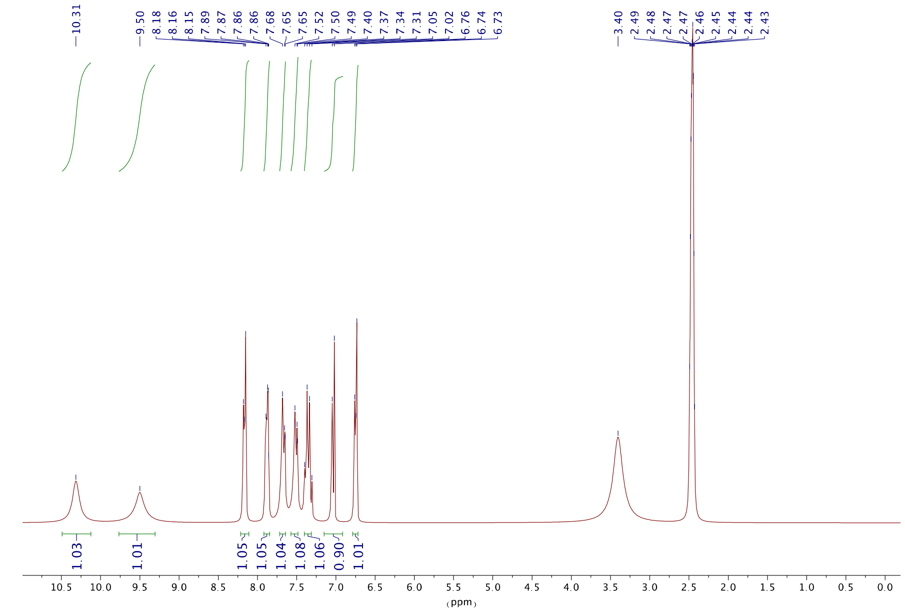
10.31
9.50
8.18
8.16
8.15
7.89
7.87
7.86
7.68
7.65
7.65
7.52
7.50
7.49
7.40
7.37
7.34
7.31
7.05
7.02
6.76
6.74
6.73
3.40
2.49
2.48
2.47
2.47
2.46
2.45
2.44
2.44
2.43
1.03
1.01
1.05
1.05
1.04
1.08
1.06
0.90
1.01
(ppm)

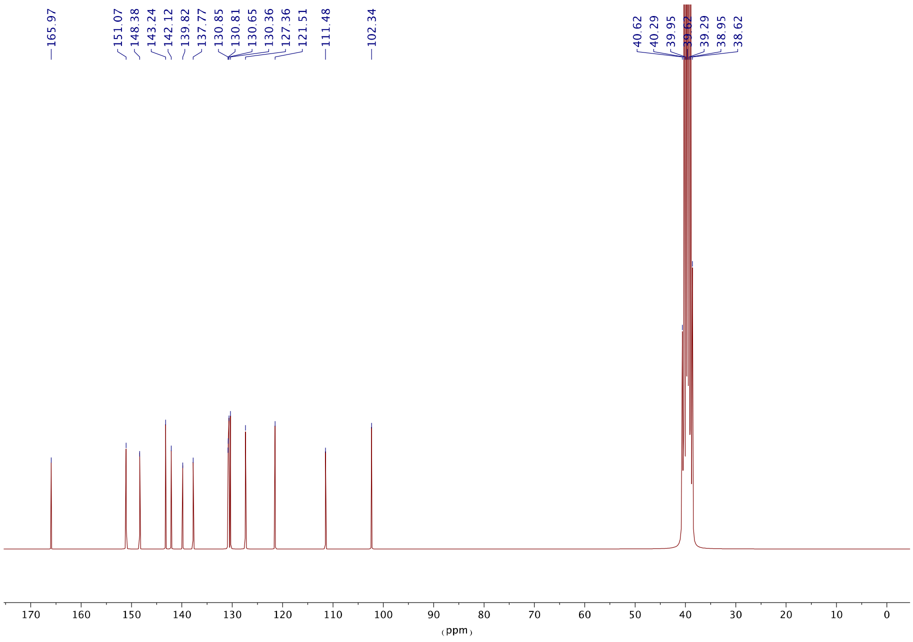
165.97
151.07
148.38
143.24
142.12
139.82
137.77
130.85
130.81
130.65
130.36
127.36
121.51
111.48
102.34
40.62
40.29
39.95
39.62
39.29
38.95
38.62
(ppm)

Compound 6

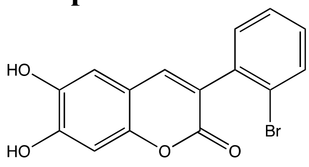
HO
HO
O
O
Br

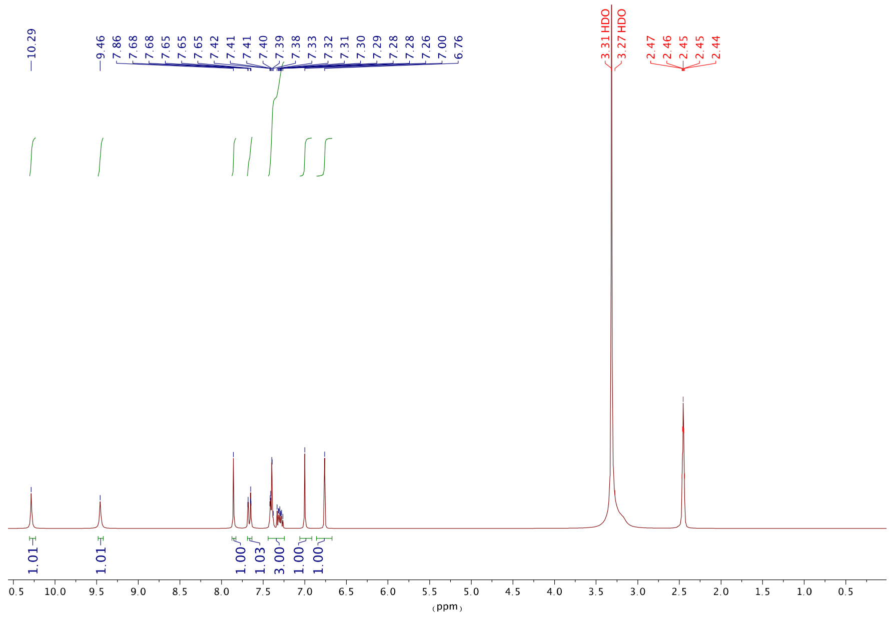
10.29
9.46
7.86
7.68
7.68
7.65
7.65
7.65
7.42
7.41
7.41
7.40
7.39
7.38
7.33
7.32
7.31
7.30
7.29
7.28
7.28
7.26
7.00
6.76
3.31 HDO
3.27 HDO
2.47
2.46
2.45
2.45
2.44
1.01
1.01
1.00
1.03
3.00
1.00
1.00
(ppm)

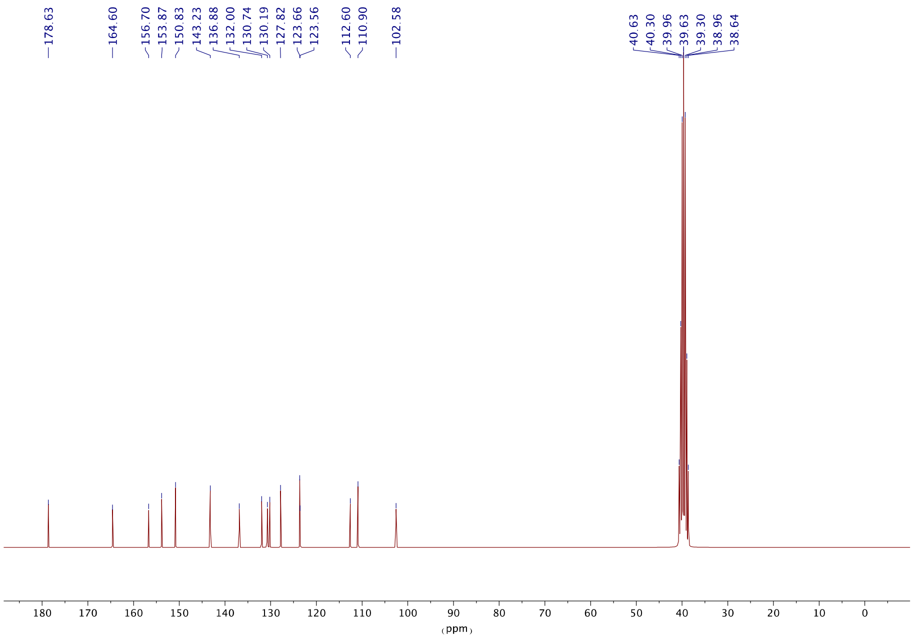
178.63
164.60
156.70
153.87
150.83
143.23
136.88
132.00
130.74
130.19
127.82
123.66
123.56
112.60
110.90
102.58
40.63
40.30
39.96
39.63
39.30
38.96
38.64
(ppm)

Compound 7

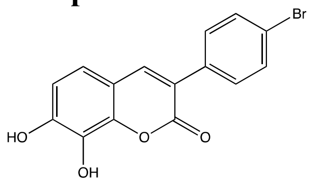
Br
HO
O
O
OH

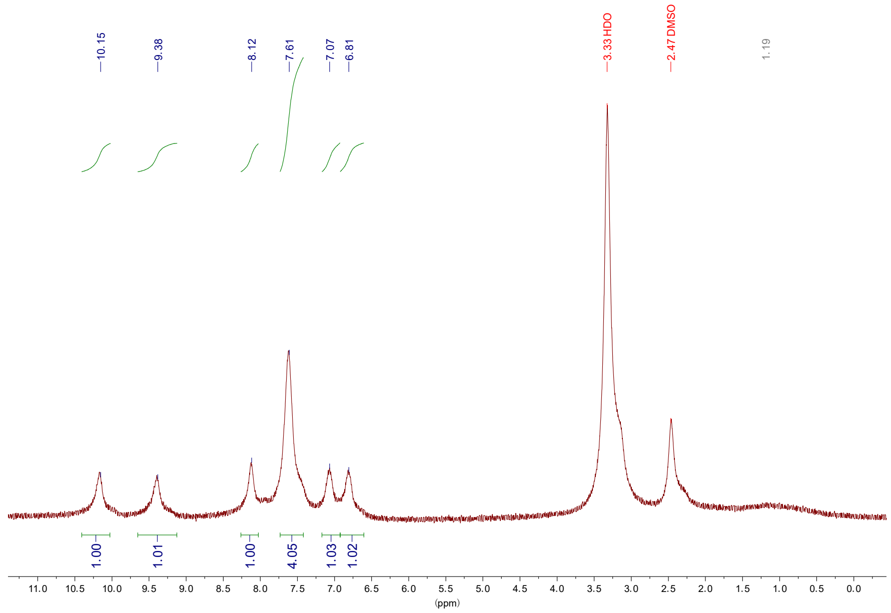
10.15
9.38
8.12
7.61
7.07
6.81
3.33 HDO
2.47 DMSO
1.19
1.00
1.01
1.00
4.05
1.03
1.02
11.0 10.5 10.0 9.5 9.0 8.5 8.0 7.5 7.0 6.5 6.0 5.5 5.0 4.5 4.0 3.5 3.0 2.5 2.0 1.5 1.0 0.5 0.0
(ppm)

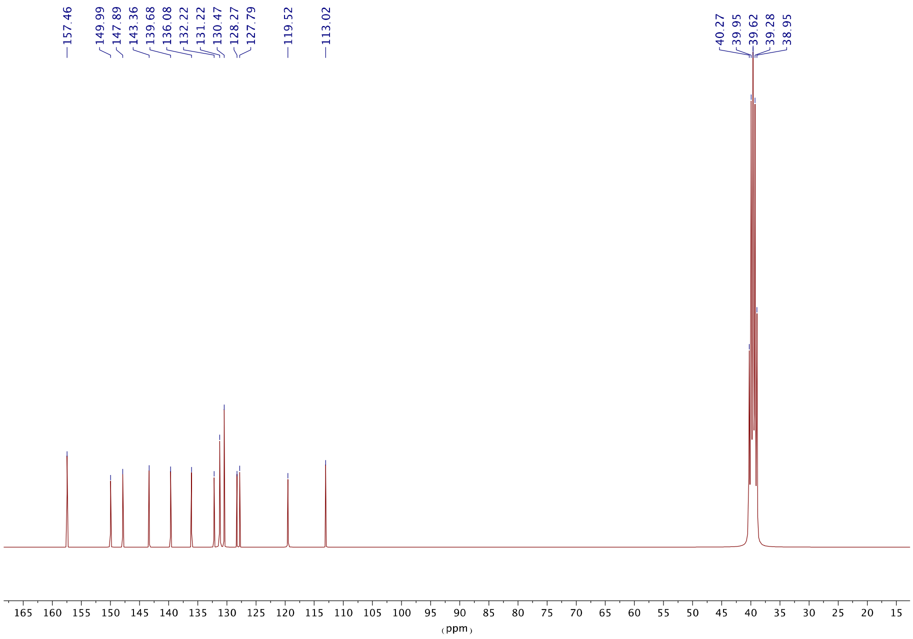
157.46
149.99
147.89
143.36
139.68
136.08
132.22
131.22
130.47
128.27
127.79
119.52
113.02
40.27
39.95
39.62
39.28
38.95
165 160 155 150 145 140 135 130 125 120 115 110 105 100 95 90 85 80 75 70 65 60 55 50 45 40 35 30 25 20 15
(ppm)

Compound 8

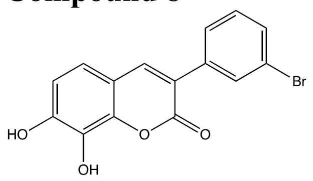
HO
OH
O
O
Br

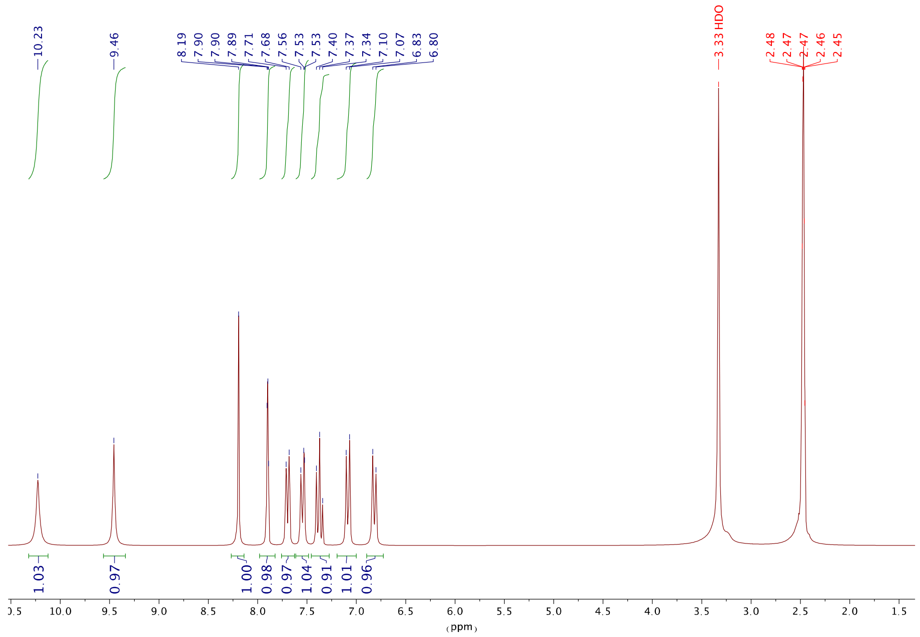
10.23
9.46
8.19
7.90
7.90
7.89
7.71
7.68
7.56
7.53
7.53
7.40
7.37
7.34
7.10
7.07
6.83
6.80
3.33 HDO
2.48
2.47
2.47
2.46
2.45
1.03
0.97
1.00
0.98
0.97
1.04
0.91
1.01
0.96
(ppm)

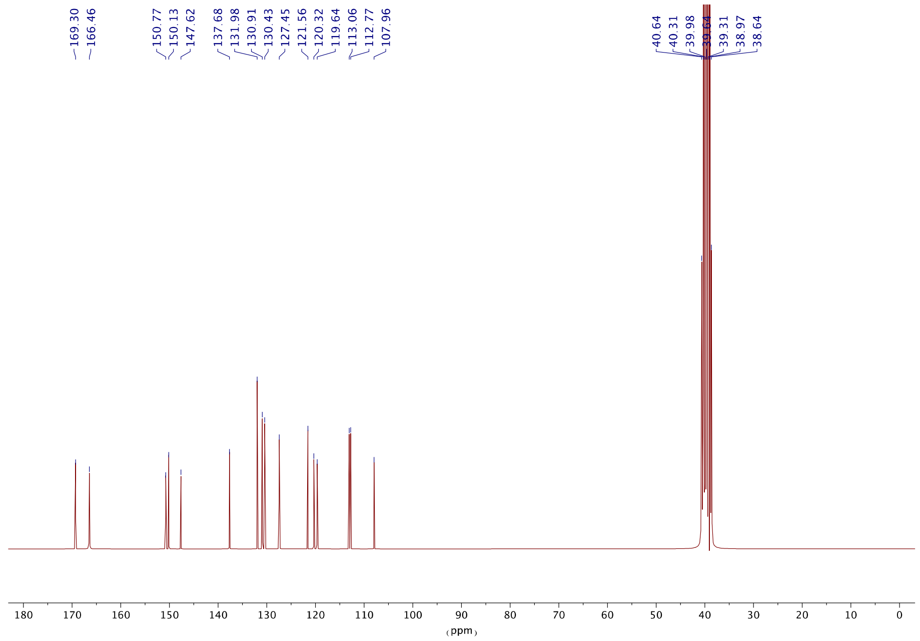
169.30
166.46
150.77
150.13
147.62
137.68
131.98
130.91
130.43
127.45
121.56
120.32
119.64
113.06
112.77
107.96
40.64
40.31
39.98
39.64
39.31
38.97
38.64
(ppm)

Compound 10

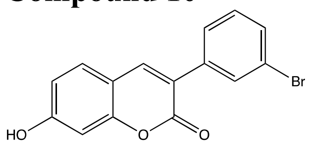
Br
HO
O
O

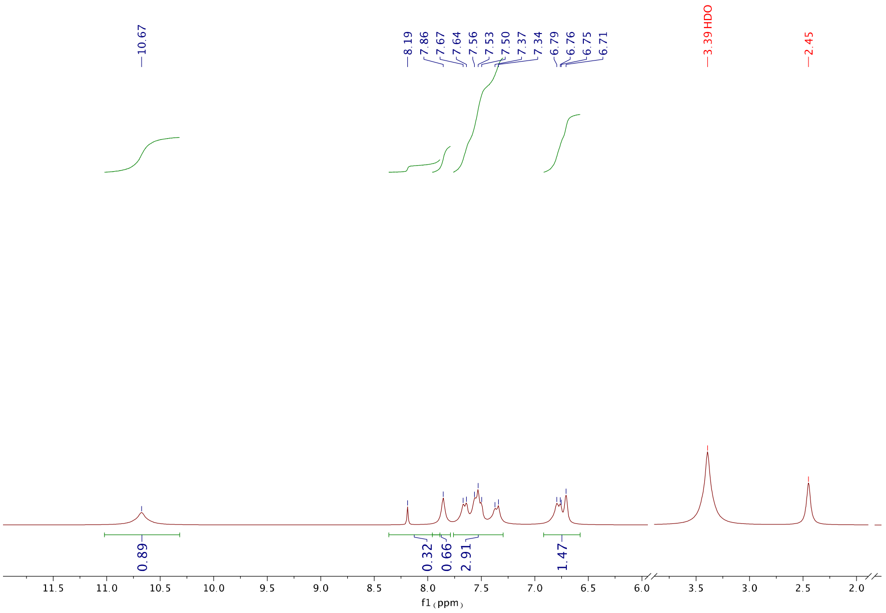
10.67
8.19
7.86
7.67
7.64
7.56
7.53
7.50
7.37
7.34
6.79
6.76
6.75
6.71
3.39 HDO
2.45
0.89
0.32
0.66
2.91
1.47
11.5 11.0 10.5 10.0 9.5 9.0 8.5 8.0 7.5 7.0 6.5 6.0 3.5 3.0 2.5 2.0
f1 (ppm)

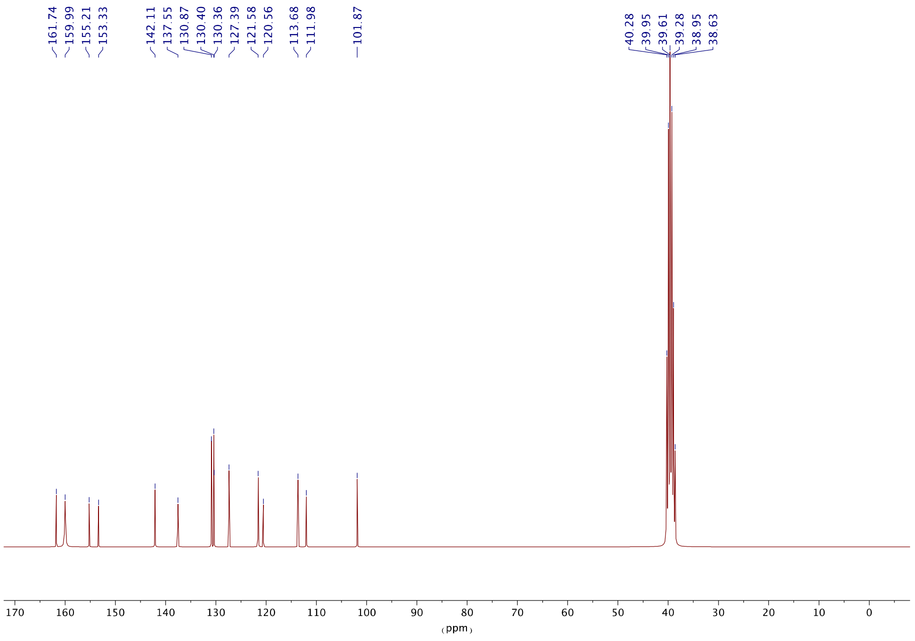
161.74
159.99
155.21
153.33
142.11
137.55
130.87
130.40
130.36
127.39
121.58
120.56
113.68
111.98
101.87
40.28
39.95
39.61
39.28
38.95
38.63
170 160 150 140 130 120 110 100 90 80 70 60 50 40 30 20 10 0
(ppm)

Compound 12

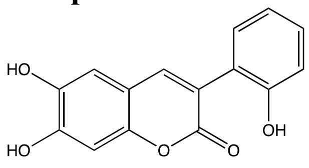

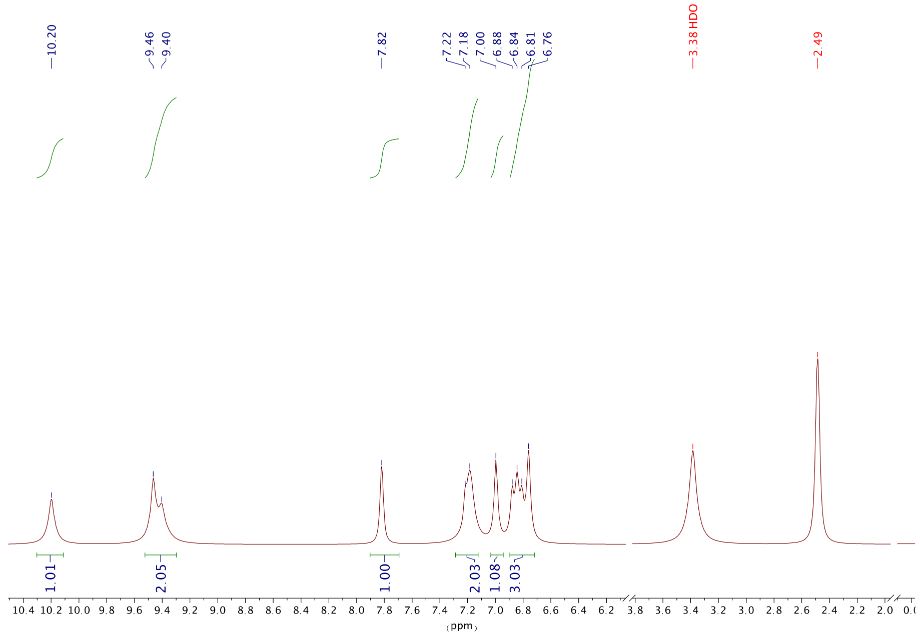

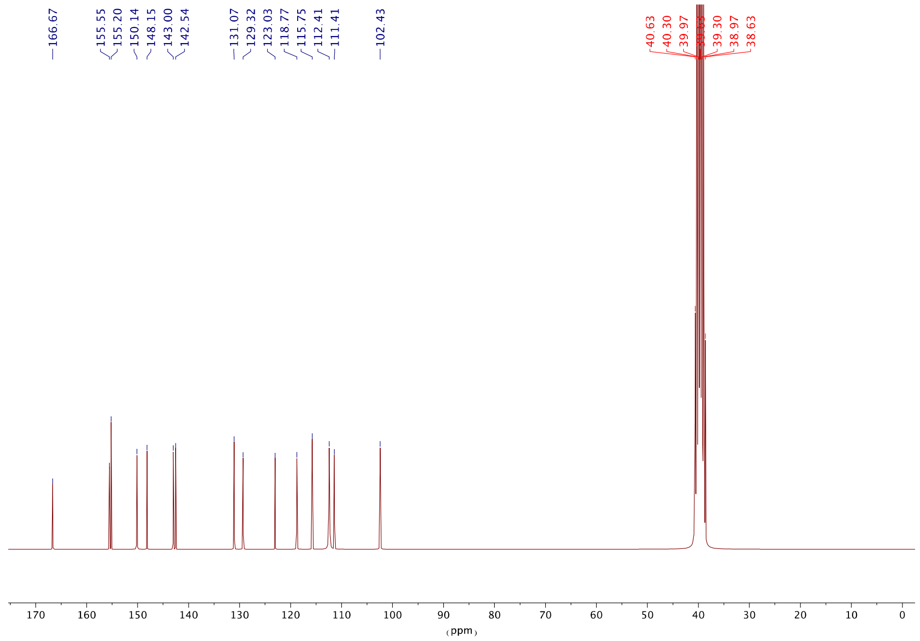

Figure S1. ^{1}H NMR and ^{13}C NMR spectra of the new compounds **2-8**, **10** and **12**.

Table S1. Enzyme inhibition of compounds at concentrations of 50 µM (tyrosinase and elastase), 100 µM (collagenase) and 200 µM (hyaluronidase).

Compound	%			
	Tyrosinase	Elastase	Collagenase	Hyaluronidase
**1**	98.3	15.6	42.6	19.0
**2**	79.2	20.3	44.3	23.2
**3**	29.3	N.I	37.1	28.5
**4**	58.3	30.5	N.I.	91.5
**5**	80.3	68.4	18.2	N.I.
**6**	25.8	23.8	38.1	13.7
**7**	9.6	15.6	N.I.	18.1
**8**	33.6	2.2	39.9	31.1
**9**	15.7	N.I	14.7	29.5
**10**	56.9	38.9	13.2	23.3
**11**	8.6	29.6	35.7	43.2
**12**	22.7	24.5	39.3	14.2
**13**	23.2	20.7	51.5	20.5
**14**	22.3	35.3	21.2	11.2
**15**	N.I	N.I	N.I.	12.7
**16**	N.I	22.8	7.9	14.9
**17**	10.0	22.4	15.6	14.0

N.I. = No inhibition
